# Syndrome of inappropriate antidiuretic hormone secretion is associated with different proton pump inhibitor use: a pharmacovigilance study

**DOI:** 10.1186/s12882-022-02818-3

**Published:** 2022-05-19

**Authors:** Mengmeng Wang, Lingjian Zhang, Min Jia, Junyan Wang, Zhiwen Shen, Shuyue Wang, Xinghui Zhang, Jing Xu, Zheng Zheng, Xuanrui Lv, Xiaoyu Zong, Hui Li, Jin Zhou, Tong Meng, Mingzhu Chen, Bin Zhao, Jian Gong

**Affiliations:** 1 Research Group of Jian Gong on Pharmacoepidemiology and Clinical Drug Evaluation, School of Life Science and Biopharmaceutics, Shenyang Pharmaceutical University, P.O.Box 88, No.103 Wenhua Road, Shenyang, 110016 P.R. China; 2 Department of Pharmacy, Chengdu Fifth People’s Hospital, Chengdu, 611130 PR China; 3 Department of Pharmacy, Peking Union Medical College Hospital, Beijing, 100730 PR China

**Keywords:** Syndrome of inappropriate antidiuretic hormone secretion, Adverse event reporting system, Pharmacoepidemiology, Clinical drug evaluation, Proton pump inhibitors

## Abstract

**Aim:**

The objective of this study was to evaluate the reported associations between the syndrome of inappropriate antidiuretic hormone secretion (SIADH) and a variety of proton pump inhibitors (PPI) through analysis of the reports extracted from the Food and Drug Administration Adverse Event Reporting System (FAERS).

**Methods:**

FAERS reports from January 2004 to March 2020 were used to conduct disproportionality and Bayesian analyses. The definition of SIADH relied on the preferred terms provided by the Medical Dictionary for Regulatory Activities. The time to onset, mortality, and hospitalization rates of PPI-related SIADH were also investigated.

**Results:**

The study identified a total of 273 reports of PPI-associated SIADH, which appeared to influence more elderly than middle-aged patients (71.1% vs. 12.5%). Women were more affected than men (48.7% vs. 41.8%). Rabeprazole had a stronger SIADH association than other PPIs based on the highest reporting odds ratio (reporting odds ratio = 13.3, 95% confidence interval (CI) = 7.2, 24.9), proportional reporting ratio (proportional reporting ratio = 13.3, χ^2^ = 113.7), and empirical Bayes geometric mean (empirical Bayes geometric mean = 13.3, 95% CI = 7.9). The median time to SIADH onset was 22 (interquartile range 6–692) days after PPI administration. PPI-associated SIADH generally led to a 2.95% fatality rate and a 79.7% hospitalization rate. The highest hospitalization death rate occurred in esomeprazole (91.2%).

**Conclusion:**

According to our findings, more attention should be paid to SIADH within the first several months after the administration of PPIs. For women older than 65 years, dexlansoprazole may reduce the incidence of PPI-associated SIADH. Nonetheless, larger epidemiological studies are suggested to verify this conclusion.

## Introduction

Proton pump inhibitors (PPI) block the activity of H^+^, K^+^-ATPase in gastric parietal cells and secretion of gastric acid caused by any stimulation [1]. Since the late 1980s when omeprazole entered the clinic, PPIs have been widely used for the treatment of a variety of acid-related conditions, such as peptic ulcer disease and gastroesophageal reflux disease [2–4]. Omeprazole, esomeprazole, pantoprazole, lansoprazole, and rabeprazole all belong to the PPI family.

Over the last decades, the increased use of PPI has been observed in many countries [5, 6]. Meanwhile, concerns have been raised regarding potential adverse reactions associated with PPI use. With the increase in the number of epidemiological studies related to PPI, evidence has emerged that PPIs are related to the occurrence of gastric neoplasia, renal disease, fracture risk, dementia, liver disease, and micronutrient deficiency [7–10]. Recent case reports indicated that the syndrome of inappropriate secretion of antidiuretic hormone (SIADH) is linked to PPIs [11–13].

Our study was motivated by the paucity of studies on PPI-related SIADH and differences between individual PPIs remain unknown. Therefore, the purpose of this retrospective study was to identify and compare SIADH related to different PPIs by leveraging the reports extracted from the Food and Drug Administration Adverse Event Reporting System (FAERS).

## Methods

### Data source

This retrospective pharmacovigilance analysis was conducted based on reports extracted from the FAERS database. FAERS is a public adverse events database that contains anonymized adverse events and medication errors reported by healthcare professionals, manufacturers, and consumers from all around the world since 1968. The reports included for this study ranging between January 2004 and March 2020 were used for retrospective inverse frequency analysis of the drugs involved. Among a total of 196,482 records were included in the full FAERS dataset, 273 were identified as reports for SIADH after PPI treatment.

### Terms for adverse events and drug identification

According to the Medical Dictionary for Regulatory Activities (version 23.0), SIADH reports secondary to PPIs were extracted from the FAERS database by applying the preferred term” inappropriate antidiuretic hormone secretion (Code: 10053198)”. Since FAERS does not use a unified drug coding system, omeprazole, esomeprazole, pantoprazole, lansoprazole, rabeprazole, dexlansoprazole, and their brand names were used to identify PPI-related records. Thus, MICROMEDEX (Index Nominum) was chosen as a dictionary for data mapping.

### Data mining

Based on Bayesian and non-proportional analyses, the reporting odds ratio (ROR), proportional reporting ratio (PRR), Bayesian confidence propagation neural network (BCPNN), and multi-item gamma Poisson shrinker (MGPS) algorithms were used to investigate the relationship between PPIs and SIADH.

ROR was first proposed by the Dutch Pharmacovigilance Center, where an adverse reaction signal is generated when the lower limit of the ROR 95% confidence interval (CI) is > 1 [14–16]. PRR along with χ^2^ are the early methods for quantitative analysis of spontaneous reporting systems. PRR > 2 and χ^2^ > 4 indicate that an adverse reaction signal is generated [17]. BCPNN is a set of methods for exploring adverse drug reaction signals established by the International Drug Monitoring Cooperation Center in 2002 [18]. The core of the BCPNN method is to calculate the information component (IC) value [14, 19–21]. IC025 represents the lower limit of the 95% two-sided CI. IC025 > 0 indicates that there is a relationship between the suspected drug and the adverse reaction. The size of the IC value reflects the strength of the connection between the suspected drug and the adverse reaction [18]. The core of the MGPS method is to calculate the empirical Bayesian geometric mean (EBGM) [22]. The calculation principle is similar to that for the IC value. Finally, the 95% CI for EBGM is obtained and the lower limit is expressed using EBGM05. EB05 > 2 serves as a prompt to generate a signal [22, 23].

In addition, the SIADH onset time, the interval between adverse event occurrence date and PPI administration start date, was evaluated for different PPIs. When the adverse event onset date was earlier than the PPI administration start date, it is considered as a default error to be excluded for further analysis.

### Statistical analysis

Descriptive analysis was used to summarize the demographic information for PPI-related SIADH. A non-parametric test was used to compare the onset time for PPI-related SIADH between different PPIs. Mortality rates between different PPIs were investigated using χ^2^ or Fisher’s exact tests as needed. The statistical significance was defined as *P* < 0.05 with a 95% confidence interval (CI). All analyses were conducted using GraphPad Prism version 8.4.3 (GraphPad Software, Inc., USA).

## Results

### Descriptive analysis

The overview of SIADH used for PPIs is shown in Table 1. There were a total of 273 reports of SIADH-related PPIs identified. Over 80% of reports were from Europe (81.3%), followed by 8.8% of cases from Asia. 85.7% of reports were submitted by healthcare professionals, accounting for the vast majority of all reports. The number of PPI-related SIADH cases has gradually increased between 2004 and 2020, peaking at 72 (26.4%) in 2019. Excluding unspecified data, women account for a larger cohort proportion than men (48.7% vs. 41.8%). Similarly, patients aged 65 and over were more affected than other patients (71.1% vs. 12.5%), where elderly (75–84 years old) individuals accounted for 30.0% of all patients. Among PPIs, the most common adverse reaction to SIADH was due to omeprazole (*n* = 122, 44.7%) followed by pantoprazole (*n* = 64, 23.4%). Dexlansoprazole was not found in any SIADH reports to be associated with PPIs. Gastroesophageal reflux disease was the main indicator of PPI (37, 17.1%), while peptic ulcer ranked second (14, 6.2%).

**Table 1:** Basic demographic and clinical information of patients with PPIs -associated SIADH (January 2004 to March 2020)

Characteristics	Reports,no(%)
**Reporting region**	
**Europe**	222 (81.3)
**Asian**	24 (8.8)
**North America**	18 (6.6)
**Oceania**	2 (0.7)
**Unspecified**	7 (2.6)
**Reporters**	
**Healthcare professionals**	234 (85.7)
**Non-healthcare professionals**	13 (4.8)
**Unspecified**	26 (9.5)
**Reporting year**	
**2019**	72 (26.4)
**2018**	33 (12.1)
**2017**	32 (11.7)
**2016**	20 (7.3)
**2015**	24 (8.8)
**2014**	13 (4.8)
**2013**	11 (4.0)
**2012**	11 (4.0)
**2011**	27 (9.9)
**2010**	7 (2.6)
**2009**	4 (1.5)
**2008**	3 (1.1)
**2007**	3 (1.1)
**2006**	4 (1.5)
**2005**	6 (2.2)
**2004**	3 (1.1)
**Sex of patients**	
**Male**	114 (48.7)
**Female**	133 (41.8)
**Unknown or missing**	26 (9.5)
**Age groups (years)**	
**<18**	2 (0.7)
**18–44**	4 (1.5)
**45–64**	28 (10.3)
**65–74**	71 (26.0)
**75–84**	82 (30.0)
**≥ 85**	41 (15.0)
**Unknown or missing**	45 (16.5)
**PPIs as suspected drugs**	
**Omeprazole**	122 (44.7)
**Lansoprazole**	43 (15.8)
**Pantoprazole**	64 (23.4)
**Rabeprazole**	10 (3.7)
**Esomeprazole**	34 (12.5)
**Dexlansoprazole**	0 (0.0)
**Indications for tumors of different sites**	
**Gastroesophageal reflux disease**	37 (17.1)
**Peptic ulcer disease**	14 (6.5)
**Gastroduodenitis**	10 (4.6)
**Functional dyspepsia**	9 (4.2)
**Epigastric pain or discomfort**	7 (3.2)
**Non-specified gastrointestinal disorders**	4 (1.9)
**Eradication of** ***Helicobacter pylori*** **infection**	4 (1.9)
**Peptic ulcer-related gastrointestinal bleeding**	2 (0.9)
**Lower abdominal discomfort**	2 (0.9)
**Esophagitis**	1 (0.5)
**Other indications**	28 (13.0)
**Unknown or missing indications**	98 (45.4)

*PPIs* Proton pump inhibitors, *SIADH* Inappropriate secretion of antidiuretic hormone

### Signals for PPI-associated SIADH

The correlation between PPIs and SIADH was evaluated according to four algorithms (Table 2). Among all PPIs, rabeprazole had the strongest relationship with SIADH (ROR 13.4; PRR 13.3; EBGM 13.3), and omeprazole ranked second (ROR 9.7; PRR 9.6; EBGM 9.4). Esomeprazole was not associated with the SIADH signal according to the resulting ROR, PRR, and EBGM values.

**Table 2:** Association of different PPIs with SIADH

Drug	N	ROR (95% CI)	PRR (χ^**2**^)	IC (IC025)	EBGM (EBGM05)
**Omeprazole**	122	9.7 (8.1, 11.6)^a^	9.6 (920.1)^a^	3.2 (2.7)^a^	9.4 (8.1)^a^
**Lansoprazole**	43	4.5 (3.3, 6.0)^a^	4.4 (113.9)^a^	2.1 (1.6)^a^	4.4 (3.4)^a^
**Dexlansoprazole**	0	0 (−)	0 (−)	0 (−)	0 (−)
**Pantoprazole**	64	6.9 (5.4, 8.9)^a^	6.9 (319.1)^a^	2.8 (2.2)^a^	6.8 (5.6)^a^
**Rabeprazole**	10	13.4 (7.2, 24.9)^a^	13.3 (113.7)^a^	3.7 (2.0)^a^	13.3 (7.9)^a^
**Esomeprazole**	34	0.6 (0.5, 0.9)	0.6 (7.6)	−0.7 (−)	0.6 (0.5)

*PPIs* Proton pump inhibitors, *SIADH* Inappropriate secretion of antidiuretic hormone, *CI* Confidence interval, *PRR* Proportional reporting ratio, *ROR* Reported odds ratio, *IC* Information component, *IC025* Lower limit of the 95% two-sided confidence interval for IC

^a^denotes significant signals

### Onset time for PPI-associated SIADH

**Fig. 1:**
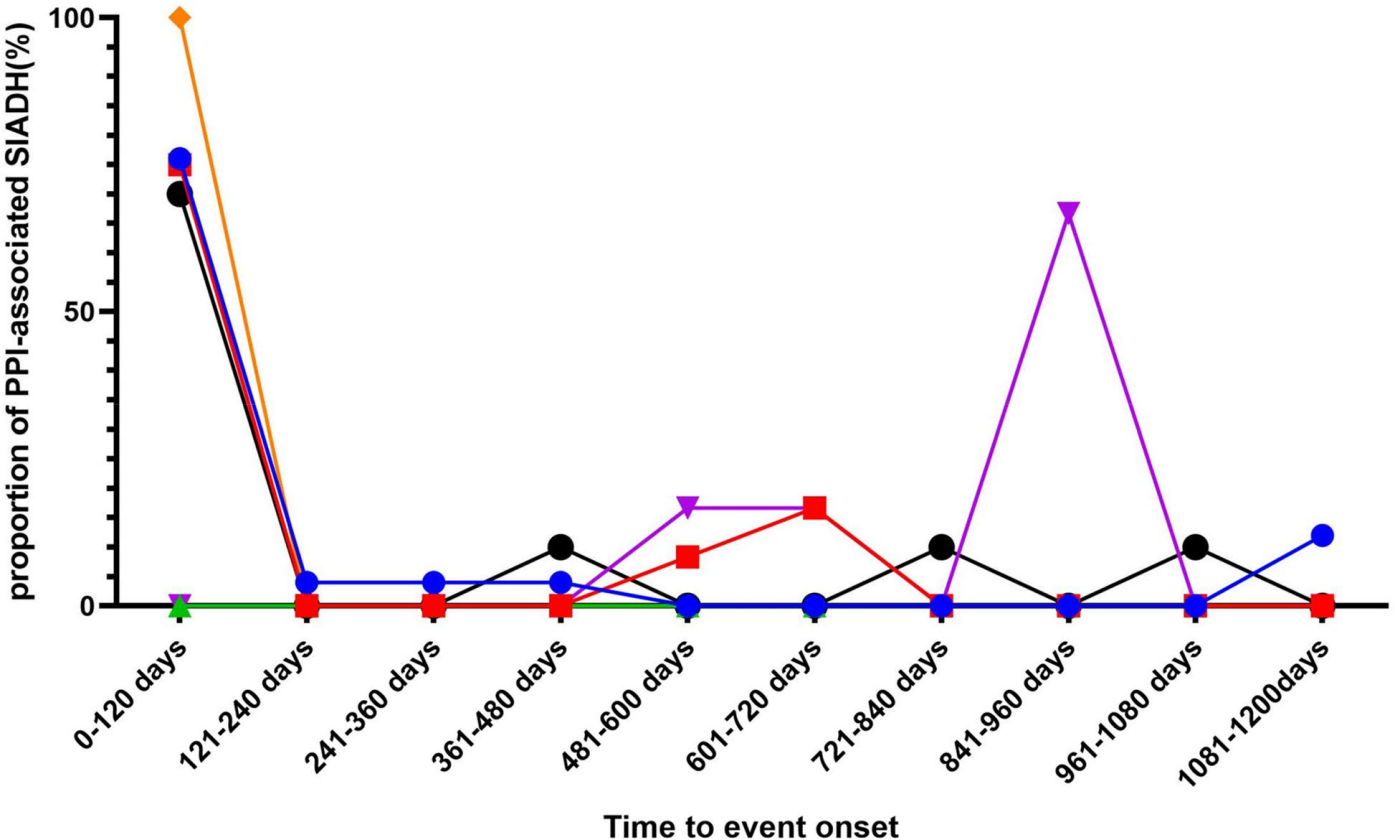
Time to event onset of SIADH following proton pump inhibitor (PPI) regimens.  omeprazole;  , esomeprazole;  ,lansoprazole;  , dexlansoprazole;  , rabeprazole;  , pantoprazole

In general, the median time to onset of PPI-associated SIADH was 22 (interquartile range [IQR] 6–692) days. Figure 1 illustrates the SIADH onset for each PPI. It is worth noting that in addition to pantoprazole, the first dose of the PPI regimen may result in the immediate occurrence of SIADH. In addition, SIADH may occur after taking pantoprazole for 841–960 days (Fig. 1). The quick SIADH onset occurred in 45.4% of all PPI-related SIADH cases, with the incidence for omeprazole, lansoprazole, pantoprazole, rabeprazole, and esomeprazole of 76.0, 75.0, 0.0, 100.0, and 70.0% respectively. There was a significant difference in the meantime to SIADH onset between pantoprazole and omeprazole (Kruskal-Wallis test, *P* = 0.03), pantoprazole and lansoprazole (Kruskal-Wallis test, *P* = 0.04), with the shortest median time of 13.5 (IQR 5–378) days for lansoprazole and the longest of 1519 (IQR 849–1519) days for pantoprazole.

### PPI-related SIADH fatality and hospitalization

**Fig. 2:**
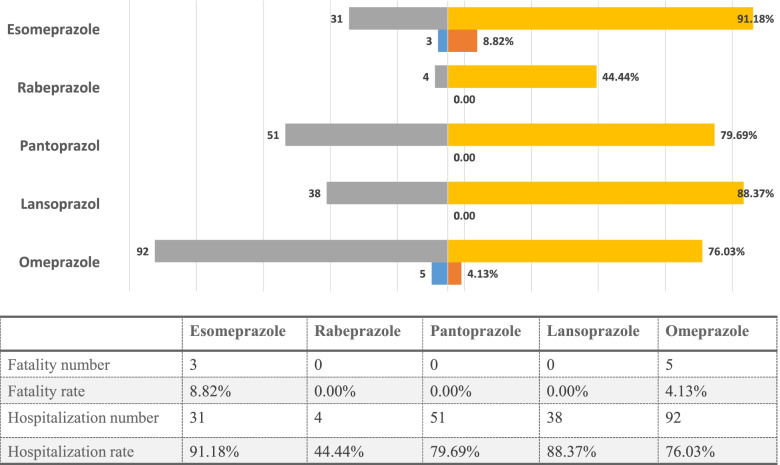
The number of fatality and hospitalization, hospitalization rates, fatality rates for PPI-associated SIADH.  , Hospitalization rate;  , Fatality rate;  , Hospitalization number;  , Fatality number

The rates of SIADH mortality and hospitalization were evaluated after various PPI treatments (Fig. 2). In total, SIADH led to a 3.0% fatality rate and a 79.7% hospitalization rate in patients administered PPIs. No significant difference was exhibited in fatality rates between different PPIs (Pearson’s chi-squared test for overall comparison, *P* = 0.20). However, a significant difference was exhibited in hospitalization rate between different PPIs (Pearson’s chi-squared test for overall comparison, *P* = 0.01). Patients with esomeprazole-related SIADH had the highest hospitalization rate (91.2%), and patients with rabeprazole-induced SIADH had the lowest hospitalization rate (44.4%).

## Discussion

SIADH is defined by euvolemic hyponatremia due to inappropriate retention of free water under the influence of antidiuretic hormone. The etiologies are divided into four major groups: tumors, drugs, central nervous system disorders, and lung disorders [24, 25]. Clinical features include water retention, increased urinary excretion, and dilutional hyponatremia [26]. As we know, Tricyclic antidepressants, selective serotonin reuptake inhibitors, chlorpromazine, and carbamazepine can cause SIADH. However, apart from case reports for drug-related SIADH, there are few reports on PPI-related SIADH [27]. Moreover, the precise mechanism of SIADH secondary to PPI is currently unclear because PPI-induced SIADH is uncommon, and it is not yet clear whether there is a difference between individual PPIs.

SIADH is considered a side effect of PPI-induced hyponatremia [28]. However, some scientists disagree with the SIADH side effect, suggesting renal salt waste instead [28, 29]. It remains unclear whether peripheral processes are responsible for the difference in PPI-induced severe hyponatremia between individual PPIs. The present research results are based on the reported adverse reactions in the FAERS database, where all SIADH diagnoses have been verified through the reports. The data and results were related to SIADH but PPI-related hyponatremia.

Although SIADH related to PPI has received clinical attention, the current related research are limited to case reports due to its extremely low incidence [11–13, 30]. No randomized controlled trials and cohort studies are identified. Therefore, we cannot make a clear conclusion regarding the safety of PPIs. The present research shows that the number of reports with an increasing trend contributed to more than 26.37% of cases in 2019. It is worth noting that SIADH was not found to be related to dexlansoprazole in the FAERS database, which is consistent with previous publications of PPI-related SIADH [11–13, 30]. A large case-control study showed that there is an association between any newly started PPI treatment except for lansoprazole and hospitalization caused by hyponatremia [27]. There are two possible reasons for the association. First, there may be fewer reports on dexlansoprazole, since it is the latest drug on the market among the six PPIs. Second, the search was limited to the articles written in English. Dexlansoprazole was developed in Japan, and FAERS data primarily focuses on the products in European and American countries.

In this literature search, healthcare professionals’ reports accounted for the largest proportion of studies, indicating that PPI-related SIADH did not attract the attention of other researchers. Literature on PPI-related SIADH mainly published as case reports indicate that the clinicians are concerned with this topic. It was also found that the elderly are more likely to develop PPI-related SIADH, indicating that age is a risk factor for SIADH. The older is a patient, the greater is the chance of incidence. The elderly usually have comorbidities, poor physical fitness, and a higher incidence of SIADH [25]. The incidence in women was also greater than that in men (48.72% vs. 41.76%), demonstrating gender differences in the adverse reactions to PPIs. Also in other large studies, women and the elderly are more affected by hyponatremia [31]. Previous studies have shown that men are more likely to suffer from peptic ulcers and gastroesophageal reflux gastritis compared to women [32–34], indicating that men have a greater chance of using PPIs. The present study shows that women are more likely to develop PPI-related SIADH, which may be due to the missing data in the FAERS database or incorrect reporting. Thus, a larger sample is needed to demonstrate the gender difference in PPI-related SIADH.

It was also found that the median time to onset of SIADH is 22 days with PPI use, suggesting that monitoring of the phenomena may be needed as soon as PPIs are started. From the perspective of the average time of SIADH occurrence, the onset time for the five PPIs ranges from half a month to three years, indicating that individual monitoring strategies need to be implemented after managing PPIs. Oral urea and vasopressin 2 antagonists are effective treatments for treating SIADH when conservative measures fail [35].

The present research has certain advantages. First, FEARS, the FDA’s adverse event reporting database provides a great opportunity to find rare adverse reactions, such as PPI-related SIADH. Second, to the best of our knowledge, this study is the first and largest real-world pharmacovigilance study comparing SIADH after using PPIs based on the FAERS database. Third, the comparison between the onset time and prognosis of SIADH is expected to serve as a reference for clinical decision-making.

Although this study has some advantages, some restrictions are specific to the FAERS database and should be considered when interpreting these results. First, the FAERS database includes some wrong information and may bias the results. Second, the available Aviation Safety Reporting System (ASRS) data only applies to patients with side effects. Despite the FAERS database has indicated it is improper to use the data to infer the prevalence of any adverse events, we must emphasize that the total number of patients being treated is unavailable, and thus some relevant statistics cannot be calculated. Third, non-proportional analysis results can only demonstrate an association but causality. Fourth, submission of a report may be biased, and it does not mean that the information included in it has been medically confirmed nor it is an admission from the reporter that the drug caused or contributed the event. They may be biased because of increased awareness affected by negative news and biased in their reporting of adverse events, which may result in spurious time trends. On the contrary, the bias may be applicable to under-reporting. There also exists a higher likelihood of adverse effects of newer drugs being reported recently, while drugs with well-known adverse effects are probably not reported as often. Formal pharmacoepidemiologic studies may be needed to overcome such biases. Although the FAERS database has some limitations, it illustrates some important aspects of PPI-related SIADH and provides clues for further studies.

## Conclusion

With the increased usage of PPI regimens in recent years, PPI-associated SIADH is on the rise. The present study found that PPIs are associated with SIADH, except for dexlansoprazole. This study also found that rabeprazole may have a stronger correlation with SIADH. Moreover, the SIADH onset time is significantly different between pantoprazole and omeprazole, pantoprazole and lansoprazole. Women older than 65 years may be more sensitive to PPI-associated SIADH. Further epidemiological research is needed to confirm these results and investigate the underlying mechanisms.

## Data Availability

The datasets generated during and analyzed during the current study are not publicly available due to the research in progress, but are available from the corresponding author on reasonable request.
